# Impact of Coastal Urbanization on Marine Pollution: Evidence from China

**DOI:** 10.3390/ijerph191710718

**Published:** 2022-08-28

**Authors:** Weicheng Xu, Zhendong Zhang

**Affiliations:** 1 School of Economics, Ocean University of China, Qingdao 266100, China; 2 Institute of Marine Development, Ocean University of China, Qingdao 266100, China

**Keywords:** urbanization, marine pollution, spatial spillover effect, nighttime light data, mechanism analysis

## Abstract

The increasing marine pollution in China’s coastal areas has seriously affected the sustainable development of the economy and the living standards of residents. It is of great significance to explore the relationship between urbanization and marine pollution in coastal areas for the sustainable development of coastal cities. Based on the marine pollution data and nighttime light (NTL) data of 46 coastal cities from 2006 to 2015, the paper discusses the impact of urbanization on marine pollution by using the generalized spatial two-stage least square method (GS2SlS), and analyzes the role of technological innovation, financial development, and human capital in the impact of urbanization on marine pollution by using the three-stage least square method (3SLS). Results show that China’s coastal marine pollution has a strong spatial spillover effect, and a U-shaped relationship exists between urbanization and marine pollution. Regional heterogeneity analysis shows that an inverted U-shaped relationship was found between coastal urbanization and marine pollution in the northern marine economic circle, while the eastern and southern marine economic circles have a U-shaped correlation. The heterogeneity of the urbanization pattern indicates that the relationship between different urbanization patterns and marine pollution in coastal areas is generally in a positive correlation stage, but the depth of urbanization occupies a dominant position. Further mechanism tests show that urbanization can effectively reduce coastal marine pollution and improve the marine environment through the technological innovation effect, financial development effect, and human capital effect.

## 1. Introduction

“Agenda 21”, released by the United Nations in 1993, announced that oceans are an essential part of the global life-support system and a valuable asset for sustainable development [1]. However, 51% of the world’s offshore eco-environment system is polluted by urban development-related activities, according to World Resources Institute statistics. In developing countries, about 90% of sewage may discharge into rivers and coastal waters without treatment [2]. Furthermore, the global economic losses resulting from marine damage amount to USD 13 billion annually [3]. Especially in China, since the reform and opening up, the economic aggregate of China’s coastal cities has grown with the urbanization process, and Shanghai and Qingdao are especially the key cities of national economic development. Based on the China Statistical Yearbook of 2020, the population urbanization rate in 11 coastal provinces in 2019 was nearly 69.66%, markedly higher than the national average of 60.60%. Nevertheless, the rapid economic growth and urbanization of coastal cities is achieved at the expense of the environment, which not only destroys the environment of the city itself, but also brings pollution beyond the self-purification capacity to the coastal waters around the city. In addition, more than 80% of all pollutants entering the ocean stem from land-based sources according to preliminary official statistics of China [4]. Based on the China Marine Ecological Environment Status Bulletin in 2019, the generally average level of water quality in China’s coastal sea area in 2019 was only 46.6%, with class I water quality declining by 7.5% year-on-year. Furthermore, the total amount of pollutants discharged from rivers into the sea remains high, and most of the marine ecosystem is in a sub-healthy or unhealthy state, and the area of green tide disasters is increasing.

To address the above problems, the report of the 18th National Congress of the Communist Party of China put forward the strategic goal of “developing the marine economy and building a maritime power” [5]. Furthermore, the high-quality development of the marine economy has great strategic significance for the utilization of marine resources, the optimization of the marine industry, and sustainable development [6]. However, the urbanization and economic development of China’s coastal cities have developed rapidly, which has also brought serious pollution to the coastal waters, especially in areas of high urbanization levels. Therefore, marine pollution has become a “stumbling block” to the sustainable development of China’s marine economy, and it is urgent to strengthen the effective treatment of marine pollution.

In the existing research on marine pollution, the aggravation of marine pollution is closely related to a variety of factors, such as the extensive method of economic growth, the lagging upgrading of industrial structure, the fuzzy property rights boundaries of marine resources, and the low level of scientific and technological innovation [7,8,9]. Moreover, the poor abilities of marine environmental governance caused by the negative externalities of marine ecological protection, the confusion of marine management systems, and the lack of social participation mechanisms in marine environmental protection are also important reasons for the increasing marine pollution [10,11]. Apart from the above elements, the impact of the accelerated urbanization of coastal cities on the marine environment cannot be ignored. The rapid development of urbanization produces combined effects and promotes the economic development of coastal cities, but it also leads to the acceleration of the manufacturing speed of urban pollutants and the intensification of marine environmental pollution [12]. From a realistic point of view, the heavily polluted areas in China’s coastal waters are also areas with a high level of urbanization. For instance, the urbanization rates of Shanghai and Zhejiang were 88.10% and 70.00% in 2019, respectively, ranking at the forefront of coastal provinces. At the same time, their coastal waters are also seriously polluted, and the proportion of the area with inferior water quality in the total sea area is at the top. The relationship between the accelerated development of urbanization and the pollution of coastal waters is not a coincidence. That said, is the increasing pollution of coastal waters in China an inevitable result of urbanization?

In fact, there may be a nonlinear relationship between coastal urbanization and marine pollution. On the one hand, the coastal areas with high urbanization levels, such as Shanghai and Zhejiang, are more seriously polluted. The rapid development of urbanization encourages coastal cities to attract a large number of favorable factors such as population, capital, and technology, thereby accelerating their own economic development. However, these cities are precisely the areas with the most serious marine pollution, which indicates that urbanization may aggravate marine pollution. On the other hand, urbanization may reduce marine pollution. With the development of urbanization, continuous technological progress, the optimization and transformation of industrial structures, and the public’s attention to the quality of marine environment, marine pollution can be decreased [13]. In addition, urbanization can produce positive externalities such as economies of scale, combined effects, and resource redistribution effects to reduce environmental damage [14]. In view of the above two aspects, urbanization has two opposite impacts on marine pollution, rather than a simple linear relationship. Hence, it is worth discussing whether there is a nonlinear relationship between urbanization in coastal areas and marine pollution.

In the process of building China into a maritime power and sustainable development, marine environmental quality is an indispensable part. Exploring the impact of urbanization on marine pollution is of great significance for promoting the green development of coastal cities and effective treatment of marine pollution. Numerous studies have discussed the causal relationship between marine pollution and urbanization [8], but there is little research on the nonlinear relationship between urbanization and marine pollution. Moreover, under the influence of natural effects such as seawater flow and economic activities such as industrial transfer, marine pollution is not only local pollution, but may have spatial correlations. This shows that when researching the relationship between urbanization and marine pollution, it is necessary to consider the spatial spillover effects of marine pollution in order to obtain more accurate results. Furthermore, the role of technological innovation, financial development, and human capital in the relationship between urbanization and marine pollution is not clear.

In view of the above reasons, the paper takes the panel data of 46 coastal cities in China from 2006 to 2015 as samples, uses the concentration data of four main marine pollutants to build a comprehensive index of marine pollution, constructs an urbanization index based on extended time series NTL data, and utilizes the spatial econometric model to explore the nonlinear relationship between urbanization and marine pollution. Next, this research further studies the impacts of technological innovation, financial development, and human capital on the relationship between them. The marginal contribution mainly includes the following three aspects: Firstly, the relationship between urbanization and the environment is extended to the field of marine ecology, and the relationship between them is discussed under the control of spatial spillover effects by using the spatial econometric model. Secondly, most literature uses the ratio of urban population to total population to represent the urbanization level, which may have statistical errors. Therefore, we construct the urbanization index based on the extended time series NTL data from 2006 to 2015. Thirdly, the 3SLS method is used to research the role of technological innovation, financial development, and human capital in the impact of urbanization on marine pollution.

The remainder of this paper is organized as follows: Section 2 provides a literature review. Section 3 introduces the materials and methods. Section 4 provides the empirical results and discussion, including spatial econometric model regression results and discussion and mechanism analysis results and discussion. This research also concludes with a conclusion and policy implications.

## 2. Literature Review

### 2.1. Research on Urbanization and Environmental Pollution

A large number of studies has discussed the relationship between urbanization and environmental pollution. In view of the relationship between urbanization and environmental pollution, the existing research mainly forms the following three viewpoints: (1) The first viewpoint is that urbanization and environmental pollution have a positive linear relationship, and that urbanization intensifies environmental pollution. Many scholars have demonstrated that the rapid development of urbanization has brought about various disasters, such as climate change, land loss, and excessive consumption of natural resources [15,16,17]. In term of different pollutants (PM_2.5_, CO_2_, etc.), many have studies found that there is a significant positive correlation between urbanization and various pollutants by using national level data [18,19,20]. For the Chinese case, Shao et al. [21] used NTL data to represent the urbanization level and found that China’s urbanization and haze present positive monotonous relationships at the national level. Liu et al. [22], using the dynamic spatial Durbin model (SDM), concluded that urbanization will increase local environmental pollution. (2) The second viewpoint is that there is a negative linear relationship between urbanization and environmental pollution, and that urbanization inhibits environmental pollution. Numerous studies have found that urbanization can reduce environmental pollution by improving the use efficiency of public transportation and other infrastructure [23], enhancing residents’ energy conservation and environmental protection behaviors [24], and by combined effect [25]. In terms of China, Yu [26] tested the environmental effects of new urbanization from four dimensions in China, and the results showed that new urbanization significantly improves energy efficiency and decreases pollution emissions. Luo et al. [27] found that promoting urbanization can be conducive to decreasing PM_2.5_ concentrations in Northwest China and Northeast China. (3) The third viewpoint is that urbanization and environmental pollution have a nonlinear relationship. Grossman and Krueger [28] found that there is an inverted U-shaped relationship between the urban economy and the ecological environment and then proposed the EKC hypothesis. After the EKC hypothesis was put forward, more and more scholars researched the nonlinear relationship between urbanization and environmental pollution. Irfan and Shaw [29] and Dong et al. [30] believed that the relationship between urbanization and environmental pollution is an inverted U-shape. However, Afridi et al. [31] found an N-shaped relationship between CO_2_ emissions and urbanization. In the case of China, Liang and Yang [32] found that urbanization and environmental pollution in China present an inverted U-shaped relationship, which is in line with the EKC hypothesis. Gan et al. [33] constructed the SDM model based on panel data of 249 cities in China and found a U-shaped relationship between urbanization and haze pollution, and they suggested that urbanization will aggravate the impact of the economy on haze pollution.

### 2.2. Research on Marine Pollution

The study of marine environments in academic circles started later than that in land regions, and marine environmental problems are complicated. Therefore, there are only a few studies on the impact of urbanization on marine pollution. Shao et al. [9] characterized marine pollution by industrial wastewater discharged directly into the sea per capita and adopted the PVAR model to analyze the interactions between marine economic growth, marine pollution, and urbanization in China. The results showed that marine economic growth and urbanization lead to marine pollution and urban expansion aggravates marine environmental damage. Yu et al. [34] explored the impact of urbanization on marine pollution based on the data of China’s coastal prefecture-level cities and found that there is a U-shaped relationship between urbanization and marine pollution.

Many scholars have also studied the relationship between other factors and marine pollution in China, which can be roughly divided into the following two aspects: (1) In terms of marine economy and pollution, Chen et al. [35] reached an inverted N-shaped relationship between the marine economy and marine pollution in China, and Peng et al. [36] concluded that there was an inverted N-shaped relationship between fishery added value and marine pollution in all China’s seas. Shao [8] believed that China’s marine economy and marine pollution have not been completely decoupled, but technological innovation can reduce marine pollution. Wang et al. [37] found that there is a phenomenon of sacrificing the marine environment to develop the marine economy in China’s coastal provinces. (2) In terms of government and marine pollution, Shen et al. [38] studied the impact of coastal local government competition on marine pollution in China, and they believed that coastal local government competition increases marine pollution, and this effect is more remarkable in high fiscal pressure groups. Jiang and Li [39] considered that the gross domestic product (GDP) assessment system of local governments exacerbates marine pollution, and the tenure of local officials and marine environmental pollution have an inverted U-shaped relationship.

In order to more intuitively explain the previous research, we established a table and selected the literature for research in China that is relevant to the topic of this paper (see Appendix A, Table A1). To sum up, many scholars have discussed the relationship between urbanization and environmental pollution, but few studies focus on the impact of urbanization on marine environmental pollution. Consequently, this research constructed a comprehensive index of marine pollution using the concentration data of four major marine pollutants based on the panel data of 46 coastal cities in China from 2006 to 2015. An urbanization index was built based on extended time series NTL data, and a spatial econometric model was used to explore the nonlinear relationship between coastal urbanization and marine pollution. Then we further researched the role of technological innovation, financial development, and human capital in this relationship.

## 3. Materials and Methods

### 3.1. Study Area

**Figure 1:**
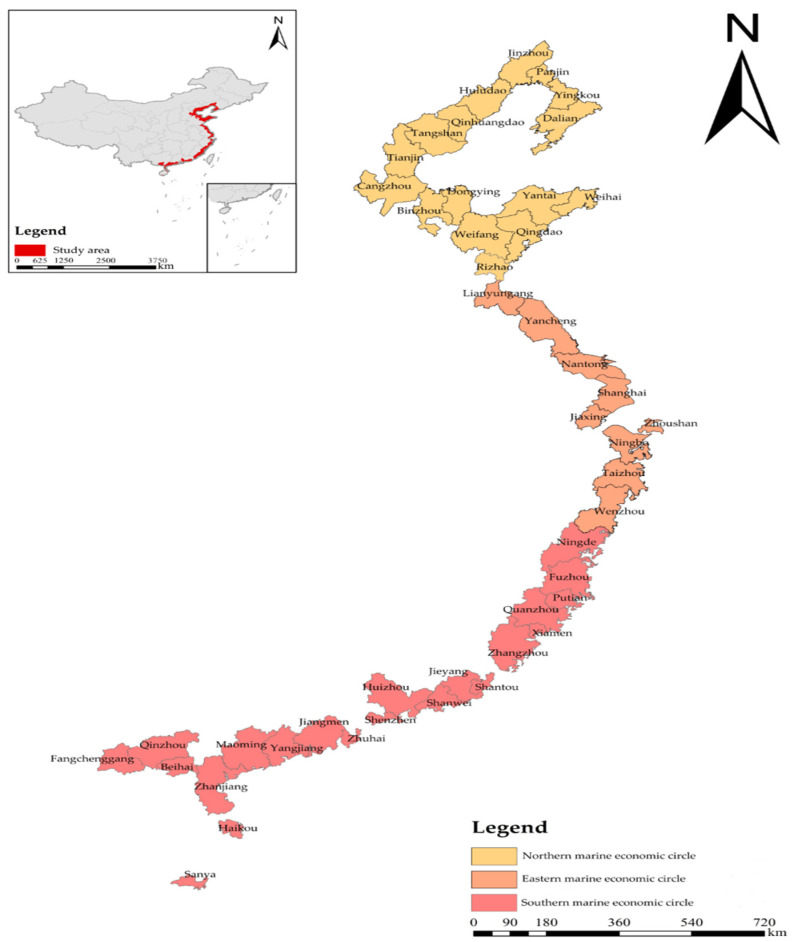
Spatial distribution of 46 coastal cities belonging to three marine economic circles.

China’s increasing marine pollution has seriously affected the green and sustainable development of coastal cities, which has become a difficult problem to be solved [8]. Therefore, in this paper, we explore the impact of urbanization on marine pollution and the mechanism by which urbanization affects marine pollution, taking China as an example. Specifically, the period 2006–2015 was chosen for this study. After excluding cities with unavailable data, we collected a total of 46 coastal cities in China for the study, including two central municipalities, four sub-provincial cities, and 40 prefecture-level cities. In addition, according to the 13th Five-Year Plan for National Marine Economic Development, we divided the coastal areas into the northern marine economic circle, eastern marine economic circle, and southern marine economic circle. The northern marine economic circle consists of the Liaodong Peninsula, the Bohai Bay, and coastal areas of the Shandong Peninsula. The eastern marine economic circle consists of coastal areas of the Yangtze River Delta. The southern marine economic circle includes Fujian, the Pearl River Estuary and its two wings, the Beibu Gulf, and coastal areas of Hainan Island (see Figure 1).

### 3.2. Model

#### 3.2.1. Spatial Econometric Model

(1) Spatial Autocorrelation Analysis Method

This paper studies the nonlinear relationship of urbanization on marine pollution based on the spatial effect. Therefore, before spatial econometric analysis, a spatial autocorrelation test is generally used to verify whether an element has a spatial spillover effect within adjacent spatial units [40]. We use Moran’s I and Moran scatterplots to test global spatial autocorrelation and local spatial autocorrelation, respectively.

According to Anselin, the Moran’s I can measure whether there is interdependence between variables in space [41]. Furthermore, Moran’s I has been widely utilized to examine spatial correlations of spatially adjacent regional units [42,43]. The specific formula is shown in Equation (1):

(1) $$I=\frac{\sum_{i=1}^{n}\sum_{j=1}^{n}w_{ij}\left(x_{i}-\bar{x}\right)\left(x_{j}-\bar{x}\right)}{S^{2}\sum_{i=1}^{n}\sum_{j=1}^{n}w_{ij}}$$

where $S^{2}=\sum_{i=1}^{n}{\left(x_{i}-\bar{x}\right)}^{2}/n$ is the sample variance, $w_{ij}$ is the (i,j) element of the spatial weight matrix, and the value of the Moran’s I is generally between −1 and 1. If it is greater than 0, it implies the existence of positive spatial autocorrelation, and if it is less than 0, it suggests the existence of negative spatial autocorrelation.

Since the Moran’s I focuses on describing whether there is spatial autocorrelation of variables in the overall distribution space, ignoring the issue of spatial correlation between regions, the spatial heterogeneity characteristics need further investigating with Moran scatterplots. In a Moran scatterplot, the region under examination is divided into four parts, namely, high–high cluster (H–H), low–high cluster (L–H), low–low cluster (L–L), and high–low cluster (H–L), which in turn correspond to quadrants I, II, III, and IV of the scatterplots, respectively. The high–high cluster means that the high value area is surrounded by the same high value areas; the low–high cluster shows that the low value area is surrounded by the high value areas; the low–low cluster means that the low value area is surrounded by areas of low value; and the high–low cluster indicates that the high value area is surrounded by low value areas.

(2) Construction of a Spatial Panel GS2SLS Model

Ehrlich and Holdren [44] put forward the IPAT model for analyzing the impact of human activities on the environment. The basic equation of the model is I=PAT, where I, P, A, and T represent environmental object, population factor, economic factor, and technological factor, respectively. The IPAT model does not allow for non-monotonic, differentially scaled changes in various influencing factors, so Dietz and Rosa [45] developed the IPAT model into the STIRPAT model, which is constructed as shown in Equation (2):

(2) $$I_{it}=\alpha P_{it}^{\beta_{1}}A_{it}^{\beta_{2}}T_{it}^{\beta_{3}}e_{it}$$

Then, in order to linearize the sequence trend and eliminate heteroscedasticity, we took the natural logarithm of both sides of Equation (2):

(3) $$lnI_{it}=ln\alpha +\beta_{1}lnP_{it}+\beta_{2}lnA_{it}+\beta_{3}lnT_{it}+lne_{it}$$

where the subscript i and t are the city and year, respectively; α is a constant term; $\beta_{1}$, $\beta_{2}$, and $\beta_{3}$ are the parameters to be estimated for each variable coefficient; and *e* is the error term.

The STIRPAT model has greater flexibility compared with the IPAT model. It can not only directly estimate the parameters of each coefficient in the model but can also decompose it and add other relevant control variables. In order to research the impact of urbanization on marine pollution, based on the STIRPAT model, this paper took marine pollution as the environmental object and added the core explanatory variable urbanization level. In addition, we also added the quadratic term of urbanization in order to explore the nonlinear relationship between urbanization and marine pollution. Moreover, considering that marine pollution may also be affected by other factors, a series of control variables was added to the model, and the following panel model was obtained:

(4) $$lnmp_{it}=\alpha_{0}+\beta_{1}lnurban_{it}+\beta_{2}{\left(lnurban_{it}\right)}^{2}+\sum_{j=3}^{10}\beta_{j}lncontrol_{it}+\varepsilon_{it}$$

where $lnmp_{it}$ is the comprehensive index of marine pollution of coastal city i in year t, $lnurban_{it}$ is the urbanization level of coastal city i in year t, $lncontrol_{it}$ is a series of control variables, $\alpha_{0}$ is the constant term, $\beta_{1}$ to $\beta_{10}$ are the estimated coefficients of variables, and $\varepsilon_{it}\;$is a random perturbation term.

Using the usual fixed effects panel model to estimate the content of this article results in some mistakes. The reason for the error is that the geographical relationship of the amount of pollution is ignored. That is, the impact of nearby cities on the city is overlooked. Therefore, we introduced the spatial lag term of marine pollution into the above panel model and constructed a spatial panel GS2SLS model to explore the impact of urbanization on marine pollution under the spatial effect.

(5) $$lnmp_{it}=\alpha_{0}+\rho W_{i}lnmp_{it}+\beta_{1}lnurban_{it}+\beta_{2}{\left(lnurban_{it}\right)}^{2}+\sum_{j=3}^{10}\beta_{j}lncontrol_{it}+\varepsilon_{it}$$

where ρ  is a spatial autoregression coefficient, and $W_{i}$ is the spatial weight matrix. We constructed a spatial adjacency matrix $\left(W_{1}\right)$ to reflect the influence of geographical factors on marine pollution, the setting principle of which is as follows: if two cities are adjacent in spatial location, the matrix element is 1; otherwise, it is 0 [46]. Moreover, the geographical distance matrix $\left(W_{2}\right)$ was also constructed for robustness testing [47]. $W_{2}$ calculates the geographical distance between each other according to the central longitude and latitude coordinates of each city.

The GS2SLS method was utilized in this study to estimate the spatial panel GS2SLS model, which uses explanatory variables and spatial lag terms as instrumental variables and estimates the spatial panel model based on the 2SLS method [48]. Since the causal relationship between urbanization and marine pollution [49] and the possible missing variables will lead to endogenous problems, the use of OLS estimation will result in parameter estimation inconsistency. However, the advantage of using the GS2SLS estimation method is that it can control the spatial spillover effect and endogenous problem of marine pollution at the same time. In the paper, the highest third-order spatial lag term was selected as the instrumental variable for spatial panel GS2SLS model regression, and the highest second-order spatial lag term was used as the instrumental variable for robustness testing [50].

#### 3.2.2. Mechanism Analysis Model

The above spatial econometric model was used to investigate the impact of urbanization on marine pollution under the spatial effect, and in the following we constructed a mechanism model to further study the mechanism by which urbanization affects marine pollution. Specifically, numerous studies have shown that economic growth can greatly promote urbanization development, which shows that economic development is the key factor to promote urbanization [51]. Therefore, we introduced Equation (6) into the mechanism model. At the same time, existing studies have shown that urbanization can influence technological innovation, financial development, and human capital and then affect environmental pollution [52,53,54]. Therefore, we introduced Equations (7) and (8) to detect the impact of the above intermediate variables on marine pollution. To sum up, we established the following mechanism model based on the method of Mao [55] to analyze the roles of technological innovation, financial development, and human capital on the impact of urbanization on marine pollution, namely, to verify whether the technological innovation effect, financial development effect, and human capital effect in the process of urbanization are favorable for reducing marine pollution and improving the marine environment.

(6) $$lnurban_{it}=a+blnpgdp_{it}+\theta \sum^{\;}x_{it}+\varepsilon_{it}$$

(7) $$m_{it}=a+clnurban_{it}+\theta \sum^{\;}x_{it}+\varepsilon_{it}$$

(8) $$lnmp_{it}=a+dm_{it}+\theta \sum^{\;}x_{it}+\varepsilon_{it}$$

where$\;lnpgdp_{it}\;$represents economic growth; $x_{it}\;$represents population density (lnpop), energy efficiency (lnee), industrial structure (lnis), government intervention (lngi), degree of openness to the outside world (lnfdi), and marine economic development (lnpgop); and $m_{it}\;$represents technological innovation ($lnti_{it}$), financial development level ($lnfin_{it}$), and human capital ($lnhc_{it}$). Furthermore, a is a constant term; b, c, and d are the estimated coefficients of variables; and $\varepsilon_{it}$ is a random perturbation term. If the coefficients b, c, and d are all significant, this indicates that the mechanism test is valid.

The 3SLS method was utilized in this study to estimate the mechanism model. For a multi equation system, if none of the equations contains endogenous values, the ordinary least squares (OLS) estimation method is consistent. Due to the endogeneity of the mechanism analysis model in this paper, the OLS estimation method produces errors. However, the 3SLS estimation method can effectively alleviate endogenous problems in panel data and obtain consistent and effective estimation results [56], so we used the 3SLS method to estimate the model.

### 3.3. Variable Description

#### 3.3.1. Explained Variable: Marine Pollution Index

Marine pollution is the core explained variable, so the measurement method is particularly important. Existing studies mostly used industrial wastewater discharged directly into the sea [8,9] or the proportion of four inferior types of seawater quality [35] to measure marine pollution. However, there are two disadvantages: first, these indicators are based on provincial level data, so there will be insufficient samples and too large of a research area in the quantitative analysis, leading to deviation of results; second, it is too one-sided to consider only the marine pollution caused by industrial wastewater, and it is too general to reflect the specific changes of marine pollution by using the proportion of the four inferior types of seawater quality. Therefore, we extracted the concentration data of four marine pollutants in the coastal waters from 2006 to 2015 from the *China Coastal Environmental Quality Bulletin* and calculated the comprehensive indicators of marine pollution (mp) through Formula (9):

(9) $$mp_{it}=\alpha_{1}in_{it}+\alpha_{2}ap_{it}+\alpha_{3}cod_{it}+\alpha_{4}oil_{it}$$

where mp expresses the comprehensive index of marine pollution; and in, ap, cod, and oil represent the concentrations of inorganic nitrogen, labile phosphate, chemical oxygen demand, and petroleum in coastal water, respectively. Moreover, $\alpha_{1}$, $\alpha_{2}$, $\alpha_{3}$, and $\alpha_{4}\;$ are their weights. We calculated the average value of the point exceedance rate of each pollutant from 2006 to 2015 so as to determine their respective weights as $\alpha_{1}=0.63$, $\alpha_{2}=0.29$, $\alpha_{3}=0.03$, and $\alpha_{4}=0.05$.

#### 3.3.2. Explanatory Variable: Urbanization Level

Most literature uses population urbanization to measure urbanization level, but some scholars believe that this method has many shortcomings [52,57]. On the one hand, urbanization reflects many complex processes such as population urbanization, land urbanization, economic urbanization, and social urbanization [58], but population urbanization alone is not enough to express the real urbanization level. On the other hand, the unreasonable statistical caliber of the urban population and the statistical error of official data will lead to the deviation of the urbanization level [59]. On the contrary, NTL data can distinguish urban areas from dark rural areas, comprehensively reflect the basic information of human activities at night, and exclude the interference of human factors [52]. In addition, many scholars have proved the rationality of NTL data to represent urbanization [60,61].

Therefore, we built the urbanization index through NTL data. First, we used the Operational Line-Scan System of the Defense Meteorology Satellite Program (DMSP-OLS) and the National Polar-Orbiting Partnership Satellite’s Visible Infrared Imaging Radiometer Suite (NPP-VIIRS) NTL data according to the method of Chen et al. [62] to obtain the extended time series NTL data. Second, we used the standard three-step method to further calibrate the NTL data to improve accuracy and time comparability [60]. Third, the urbanization indicators (urban) were constructed from two aspects, namely, the depth of urbanization ($urban1_{i}$) and the breadth of urbanization ($urban2_{i}$). The calculation is as follows:

(10) $$urban1_{i}=\frac{\sum_{j=1}^{63}DN_{j}}{N\times 63}\times n_{j}$$

(11) $$urban2_{i}=\frac{A_{N}}{A}$$

where $DN_{j}$ is the gray value in level  j of city i; variable $n_{j}\;$ is the number of pixels of level j; and *N* is the total number of pixels in city  i. Therefore, $urban1_{i}\;$ represents the average nighttime light intensity in city i. Variables $\;A_{N}\;$ and A represent the area of all light pixels of city  i and the total area of city i, respectively. Hence, $urban2_{i}$ represents the night lighting scale of city  i. Finally, the urbanization level is obtained by multiplying ($urban1_{i}$ and $urban2_{i}$: $urban_{i}=urban1_{i}\times urban2_{i})\;$ [63]. In addition, we use $urban_{i}=w_{1}urban1_{i}+w_{2}urban2_{i}\;\left(w_{1}=0.8,\;w_{2}=0.2\right)\;$ to recalculate the urbanization level for robustness testing [64].

#### 3.3.3. Control Variables

In order to accurately study the impact of urbanization on marine pollution, at the same time, for alleviating the endogenous problem caused by missing variables, we selected a series of control variables with reference to relevant studies [52,65]. The specific control variables were: economic growth (pgdp), calculated by per capita GDP of each city and where the quadratic term of economic growth is introduced into the model according to the EKC hypothesis; population density (pop), calculated by population per square kilometer of the whole city; energy efficiency (ee), measured with per unit GDP of energy consumption of each city; industrial structure (is), measured with the proportion of added value of the city’s secondary industries to GDP; degree of openness to the outside world (fdi), represented by the proportion of the city’s actual utilized foreign direct investment (FDI) in GDP; government intervention (gi), calculated by fiscal expenditure to GDP; and marine economic development (pgop), measured with per capita GOP of provinces instead due to the lack of gross ocean product (GOP) data of each city.

#### 3.3.4. Intermediate Variables

In order to research the impact of the technological innovation effect, financial development effect, and human capital effect on marine pollution in the process of urbanization, we selected three intermediate variables [52,53]. The intermediate variables were: technological innovation (ti), expressed by the urban innovation index, which uses the patent renewal model to estimate the average value of each type of patent; financial development level (fin), measured with the equation $fin=\varphi_{1}fin1+\varphi_{2}fin2\;$($\varphi_{1}=0.5,\;\varphi_{2}=0.5$), where financial scale (fin1) is calculated by the ratio of total deposits and loans of financial institution in cities to GDP, and financial efficiency (fin2) is calculated by the ratio loans of financial institutions to deposits; and human capital (hc), measured by the proportion of the city’s college students in the total population.

Missing data were supplemented by the mean interpolation method. All variables were taken as the logarithms, and currency variables were adjusted to 2006 constant prices using the corresponding price indices. Descriptive statistics are shown in Table 1.

### 3.4. Data Sources

Since marine pollutants data are only published from 2006 to 2015, the data used were panel data of 46 coastal cities in China from 2006 to 2015, which were collected from the *China Coastal Environmental Quality Bulletin*. The NTL data was released by the National Oceanic and Atmospheric Administration (NOAA). Population density, GDP per capita, the proportion of secondary industry in GDP, energy consumption, and FDI and fiscal expenditure were from the *China City Statistical Yearbook and* *Statistical Bulletin*. GOP data were sourced from the *China Marine Statistical Yearbook*. In the mechanism test, the urban innovation index was from the *China Urban and Industrial Innovation Report 2017* published by Fudan University in China, and deposits and loans of financial institutions and the proportion of college students in the total population were collected from the *China City Statistical Yearbook*.

## 4. Empirical Results and Discussion

### 4.1. Spatial Econometric Model Regression Results and Discussion

#### 4.1.1. Spatial Autocorrelation Test Results and Discussion

The spatial correlation of marine pollution needed to be tested before the spatial regression. In this section, we first used the Moran’s I to test the global spatial autocorrelation of marine pollution, and then we used the Moran scatterplot to investigate the local spatial characteristics and the types of spatial clusters of marine pollution, and then we finally discussed their results.

Table 2 presents the results of global Moran’s I of marine pollution in 46 prefecture-level coastal cities in China from 2006 to 2015, tested by a spatial adjacency matrix. As can be seen from the values reported in Table 2, except for 2008, Moran’s I in other years all passed the 1% significance test. Furthermore, Moran’s I in all years were positive, indicating that there was a significant positive spatial correlation of marine pollution in China’s coastal areas.

**Figure 2:**
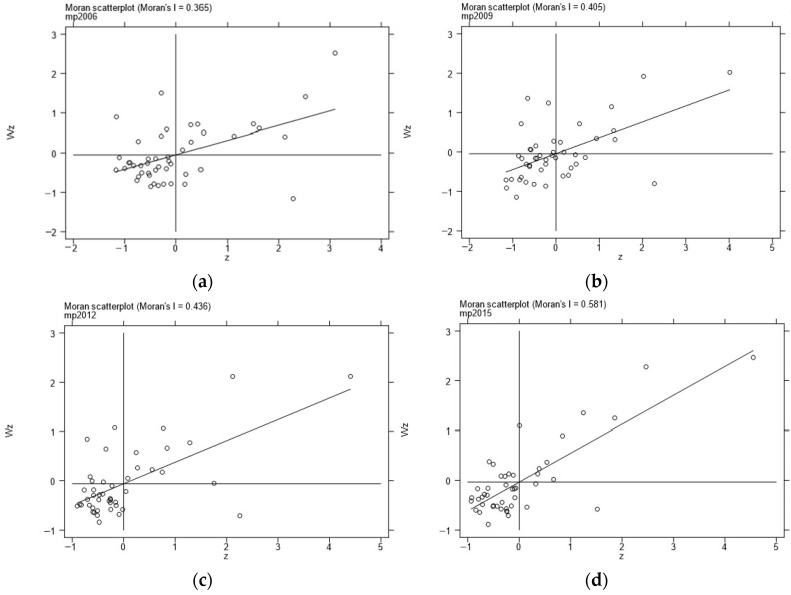
Moran scatterplots of marine pollution: (**a**) Moran scatterplot of mp in 2006; (**b**) Moran scatterplot of mp in 2009; (**c**) Moran scatterplot of mp in 2012; (**d**) Moran scatterplot of mp in 2015.

Figure 2 shows the Moran scatterplot of Chinese marine pollution values in four representative years: 2006, 2009, 2012, and 2015. From the figures, data showed that most coastal cities in different years were in the first and third quadrants, presenting the distribution characteristics of HH clusters or LL clusters; that is, the high marine pollution area was surrounded by the same high marine pollution areas, and the low marine pollution area was surrounded by the areas of low marine pollution. This result indicated that there is a spatial spillover phenomenon of marine pollution in coastal cities. In conclusion, the results of the Moran’s I and the Moran scatterplot show that there is a positive spatial autocorrelation in China’s marine pollution.

#### 4.1.2. Spatial Panel GS2SLS Model Regression Results and Discussion

The spatial panel GS2SLS model regression results are shown in Table 3. The four columns are all regression results based on the GS2SLS method under the spatial adjacency matrix. Columns (1) and (2) are listed as only considering the impact of core explanatory variables and STIRPAT model control variables (lnpop, lnpgdp, ${\left(lnpgdp\right)}^{2},\;lnee,\;lnis)$ on marine pollution. Columns (3) and (4) are the results of adding all control variables. The Hausman tests of columns (1) and (2) in Table 3 passed the significance test, indicating that the fixed-effects model should be used. However, the Hausman tests of columns (3) and (4) failed the significance test, showing that a random-effects model should be used.

The coefficients of the spatial lags of marine pollution ($W_{1}*lnmp$) in Table 3 are all significantly positive at the 5% significance level, suggesting that marine pollution has a significant spatial spillover effect. This result is similar to the conclusion of Liu et al. on China’s environmental pollution research [22]. Specifically, due to the existence of natural geographical factors such as sea water flow, the marine pollution problem of a coastal city will inevitably be affected by its spatial adjacent region. At the same time, economic factors such as industrial transfer and trade exchange brought about by regional economic development differences further deepened the spatial linkage of marine pollution problems among coastal cities [50]. To sum up, the marine pollution of coastal cities is characterized by being bound together for good or ill. Hence, coastal cities need to cooperate and conduct joint prevention and control in the process of marine pollution governance so as to better solve the marine pollution problem [22].

**Figure 3:**
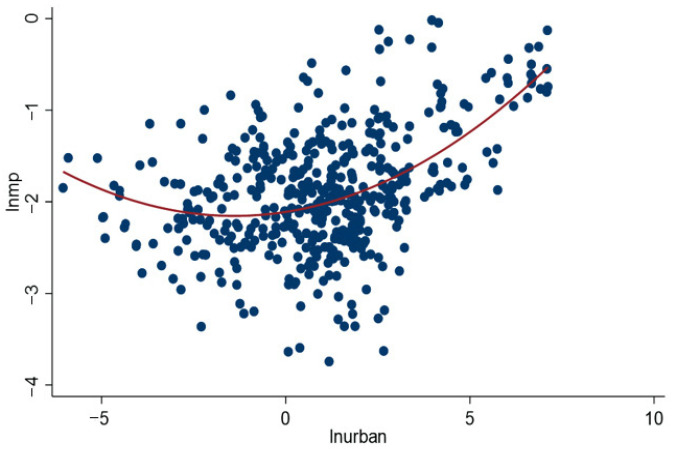
Nonlinear scatterplot of urbanization and marine pollution.

**Figure 4:**
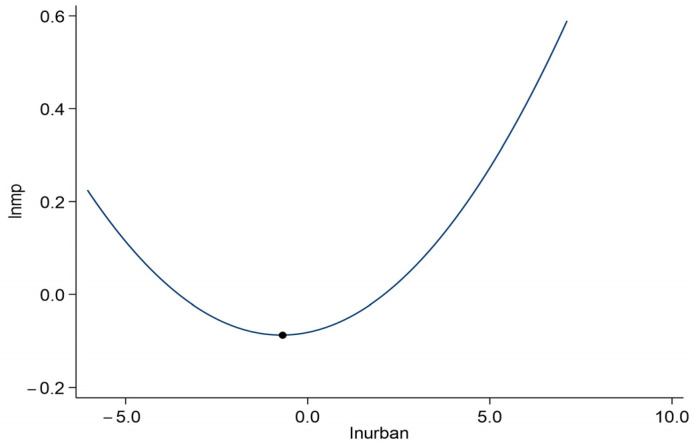
U-shaped curve between urbanization and marine pollution.

The coefficient of quadratic term of urbanization (${\left(lnurban\right)}^{2})$ in Table 3 column (4) is significantly positive at the 1% significance level, demonstrating that there is a U-shaped relationship between urbanization and marine pollution, which indicates that before the inflection point, the promotion of urbanization will improve marine pollution, but once the inflection point is crossed, the development of urbanization will aggravate marine pollution. This result is in line with that of Yu et al. [34]. To directly reflect this result, we drew the scatter diagram of marine pollution and urbanization (Figure 3) and the U-shaped curve (Figure 4). A possible reason for this is that in the early stage of coastal areas urbanization, the development of urbanization promotes the economic transformation of coastal cities, the continuous optimization and upgrading of industrial structure [52], and the improvement of people’s demand for environmental quality. At the same time, manufacturers within urban areas share all kinds of cleaner production technology and marine environment treatment equipment to boost the marine environment governance efficiency [34]. Moreover, in the process of urbanization, positive externalities such as combined effects and scale effects further reduce marine environmental pollution [14], which makes the urbanization development in coastal areas improve the marine environmental quality. However, with the continuous promotion of urbanization, the government blindly pursues the goal of urbanization in the process of coastal urbanization [38]. The population density and land use intensity of coastal cities are increasing, resulting in environmental congestion [66]. Furthermore, the land area used for greening is reduced, and the production and demand exceed the normal level, inducing a large amount of energy consumption [31]. As a result, the manufacturing speed and emission intensity of various pollutants increase continuously, and the self-purification pressure of coastal waters rises constantly, and marine pollution increases.

By calculating the inflection point of the U-shaped curve between urbanization and marine pollution, we found that the urbanization level of more than 60% of coastal cities crossed the inflection point and entered the upward stage of the U-shaped curve in 2006. In 2015, only three coastal cities (Huludao, Fangchenggang, Qinzhou) had not yet crossed the inflection point, and more than 90% of coastal cities were in the period when urbanization intensified marine pollution. From the analysis results, it shows that China’s current urbanization development path has far more promotion effects on marine pollution than inhibitory effects, so urbanization promotion will increase marine pollution.

The results of the control variables can be obtained from column (4) of Table 3. The coefficients of economic growth (lnpgdp) and the quadratic term of economic growth (${\left(lnpgdp\right)}^{2}$) were significant at the level of 1%. Economic growth (lnpgdp) had a negative impact on marine pollution, while the quadratic term of economic growth (${\left(lnpgdp\right)}^{2}$ had a positive impact on marine pollution. This implies that the relationship between economic growth and marine pollution does not satisfy the traditional EKC hypothesis, but there exists a U-shaped curve, so marine pollution has not been “decoupled” from economic growth [67]. Population density (lnpop) had a significant positive impact on marine pollution, and the increase of population density leads to the increase of various demands and excessive consumption of energy [68], which aggravates marine pollution. The impact of energy efficiency (lnee) on marine pollution was positive; that is, the improvement of energy efficiency will increase marine pollution, which suggests the existence of energy rebound effects [69] in coastal cities of China. The coefficient of degree of openness to the outside world (lnfdi) was significantly positive, indicating that FDI exacerbates marine pollution. The performance of FDI in coastal cities was consistent with the “pollution heaven” hypothesis [70]. Government intervention (lngi) had a negative impact on marine pollution, showing that government intervention is conducive to the reduction of marine pollution. Namely, the government forces enterprises to internalize environmental costs into product costs through economic means such as taxation and subsidies, thereby reducing marine pollution [71]. The coefficients of industrial structure (lnis) and marine economic development (lnpgop) were positive but did not pass the significance test.

#### 4.1.3. Robustness Checks

To test the robustness of the spatial GS2SLS model regression results, we performed robustness tests by replacing urbanization indicators, displacing the spatial weight matrix, replacing instrumental variables, and deleting samples from Shanghai and Tianjin [72]. The results of the robustness tests are shown in Table 4. The Hausman tests of columns (3) and (4) in Table 4 passed the significance test, indicating that the fixed-effects model should be used. However, the Hausman tests of the remaining columns failed the significance test, suggesting that a random-effects model should be used. Therefore, we focused on the results of columns (2), (3), (6), and (8).

The coefficients of the spatial lags of marine pollution were all significantly positive at the 1% significance level, indicating that marine pollution still has a significant spatial spillover effect. The spatial spillover effect of marine pollution is mainly caused by natural geographical factors and economic factors [50]. The coefficients of the quadratic term of urbanization were still significantly positive at the 1% significance level, demonstrating that the U-shaped relationship between the core explanatory variable of urbanization and marine pollution is robust. This shows that urbanization reduces marine pollution first, and increases marine pollution after the inflection point, which adds new evidence for the U-shaped relationship between urbanization and environmental pollution [33]. It could be observed that the spatial panel GS2SLS model regression results had strong robustness.

#### 4.1.4. Heterogeneity Analysis

(1) Heterogeneity Test Based on Different Regions

There are great differences in marine pollution and urbanization level in various coastal areas, so it is necessary to consider whether the relationship between urbanization and marine pollution has regional heterogeneity. Hence, we classified 46 coastal cities according to three marine economic circles, as shown in Figure 1. Table 5 reports the regression results for the above three regions using the GS2SLS method under the spatial adjacency matrix. According to the results of the Hausman test, the northern marine economic circle failed the test, so the random-effects model should have been selected. Both the eastern and southern marine economic circles passed the test; thus, the fixed-effects model was more desirable.

Table 5 column (2) shows that the coefficient of the spatial lag of marine pollution in the northern marine economic circle passed the 1% significance level test, but it was negative, indicating that the marine pollution of the northern marine economic circle had a significant spatial negative correlation feature. In other words, the areas with more serious marine pollution are adjacent to the areas with less pollution, and there is a cluster effect. In the column (3), the coefficient of the spatial lag of marine pollution in eastern marine economic circle was significantly positive at the 1% significance level, showing that marine pollution of the eastern marine economic circle had a positive spatial spillover effect. However, in column (5), the coefficient of the spatial lag of marine pollution in the southern marine economic circle did not pass the significance test, which means that it may not have had obvious spatial correlation.

**Figure 5:**
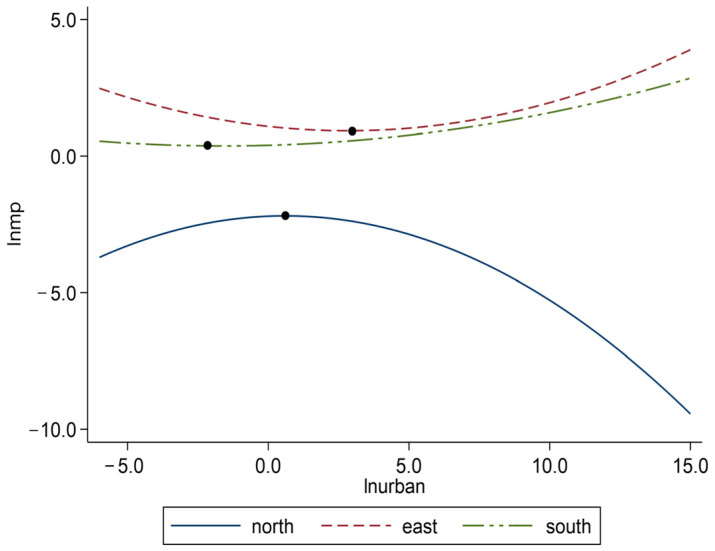
The curves between urbanization and marine pollution in different regions.

As shown in Table 5 column (2), the coefficient of the quadratic term of urbanization in the northern marine economic circle was significantly negative, suggesting that there is an inverted U-shaped relationship between urbanization and marine pollution in the northern marine economic circle, which indicates that before the inflection point, the promotion of urbanization will increase marine pollution, but once the inflection point is crossed, the development of urbanization will decrease marine pollution. However, in columns (3) and (5), the coefficients of the quadratic term of urbanization in eastern and southern marine economic circles were significantly positive, indicating that the relationship in the eastern and southern marine economic circles is still U-shaped. We plotted the relationship between urbanization and marine pollution in the northern, eastern, and southern marine economic circles (Figure 5). Figure 5 shows that with the development of urbanization, marine pollution in the eastern marine economic circle increased faster than that in the southern marine economic circle, while the continuous development of urbanization in the northern marine economic circle will eventually decrease marine pollution. Compared with the northern marine economic circle, the urbanization inhibitory effect of the eastern and southern marine economic circles did not exceed the promotion effect, or the positive externalities of urbanization had not been sufficiently exerted. A possible reason for this is that in the past two decades, the rapid economic development and the growth of population and industry in the eastern and southern marine economic circles have led to excessive urban expansion [73,74], further resulting in a large amount of energy consumption and environmental congestion, which aggravates marine pollution. Moreover, although the marine pollution in the northern marine economic circle decreased with the development of urbanization, the eastern and southern marine economic circles with serious pollution still showed a stage of increasing marine pollution, so the task of marine pollution control is quite arduous in China.

(2) Heterogeneity Test Based on Urbanization Patterns

China’s coastal cities have different development conditions, so the urbanization promotion patterns are distinct. In that way, do different urbanization patterns have different impacts on marine pollution? Therefore, we divided the urbanization patterns into the urbanization depth mode and the urbanization breadth mode. Moreover, among the three marine economic circles, the eastern marine economic circle is the area with the highest urbanization level and the most serious marine pollution. Hence, we analyzed the impacts of different urbanization patterns on marine pollution in national coastal cities and the eastern marine economic circle, respectively.

Table 6 shows the regression results of different urbanization patterns of national coastal cities using GS2SLS method under the spatial adjacency matrix. Columns (1), (2) and (3), (4) are the estimation results of the impacts of urbanization depth mode and urbanization breadth mode on marine pollution of coastal cities in China, respectively. In terms of Hausman test results, the random-effects model should be selected for the urbanization depth mode, while the urbanization breadth mode should choose the fixed-effects model. Based on the results in columns (2) and (3), the spatial lag terms of the two urbanization patterns were both significantly positive, indicating that marine pollution in the two patterns still had a positive spatial spillover effect.

**Figure 6:**
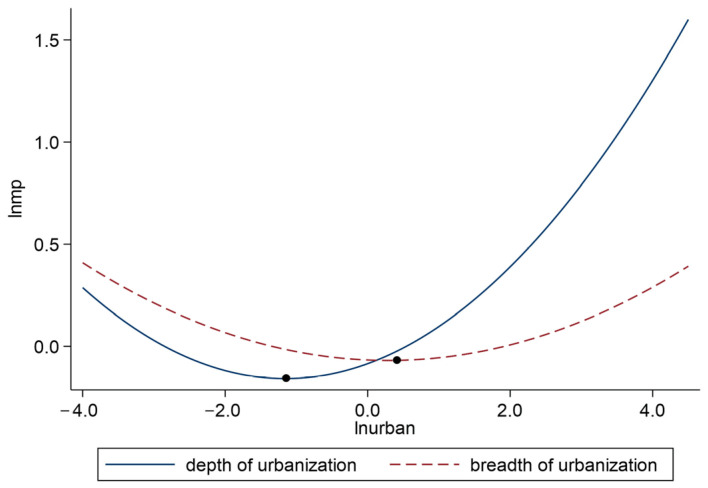
The curves of urbanization patterns and marine pollution in national coastal cities.

Figure 6 shows the curves of different urbanization patterns and marine pollution in national coastal cities. According to the coefficients of ${\left(lnurban1\right)}^{2}$ and ${\left(lnurban2\right)}^{2}$ in column (2) and (3) of Table 6 and Figure 6, there was a U-shaped relationship between two urbanization patterns and marine pollution, but the influence degree of two modes on marine pollution was different. The curve of urbanization depth mode was steeper than that of the urbanization breadth mode. Moreover, the inflection point of the urbanization depth mode was more advanced. In other words, in the early stage of urbanization, the urbanization depth mode is more conducive to reducing marine pollution than the breadth mode, and once the inflection point is crossed, the depth mode will also be more intense in enhancing marine pollution. This shows that the urbanization depth mode has a greater impact on marine pollution than the breadth mode. A possible reason for this is that although the geographical area of China’s coastal areas is small, they bear a large number of population and industries, which forces coastal cities to tend to develop the urbanization depth model. Moreover, with the development of the urbanization depth mode, the continuous improvement of the degree of agglomeration impedes the capacities of limited resources and space to carry the ever-increasing production of enterprises. Thus, a crowding effect could be produced, curbing the improvement of marine pollution [66]. According to the inflection points, only three coastal cities (Huludao, Fangchenggang, Qinzhou) have not crossed the inflection point of the urbanization depth mode, and only three coastal cities (Zhoushan, Fangchenggang, Qinzhou) have not crossed the inflection point of the urbanization breadth mode. Generally speaking, the urbanization level of most coastal cities has crossed the inflection point and entered the U-shaped curve rising stage under different urbanization patterns.

The regression results of different urbanization patterns of the eastern marine economic circle estimated under the spatial adjacency matrix using GS2SLS method are reported in Table 7. Since the quadratic term of the eastern urbanization breadth mode was not significant, the cubic term of the urbanization breadth mode was added. Columns (1) and (2) are the estimated results of the impact of urbanization depth mode on marine pollution, and columns (3)–(6) are the estimated results of the impact of urbanization breadth mode on marine pollution. Hausman test results show that columns (1) and (2) failed the Hausman test, so the random-effects model should be used. Columns (3)–(6) passed the Hausman test, so the fixed-effects model should be applied. Therefore, we focused on the regression results of columns (2) and (5). For the two urbanization patterns in the eastern marine economic circle, the coefficients of the spatial lag of marine pollution were significantly positive, indicating that there was also a positive spatial spillover effect.

**Figure 7:**
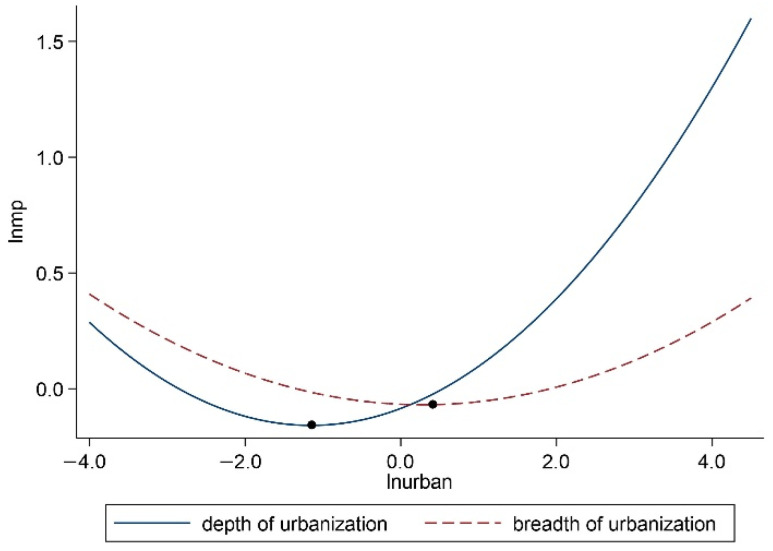
The curve of urbanization patterns and marine pollution in eastern marine economic circle.

According to the coefficients of ${\left(lnurban1\right)}^{2}$ and ${\left(lnurban2\right)}^{3}$ listed in columns (2) and (5), we plotted the curves of different urbanization patterns and marine pollution in the eastern marine economic circle. In terms of Figure 7, in the eastern marine economic circle, the impact of urbanization depth mode on marine pollution presented a U-shaped relationship consistent with the above research. However, the urbanization breadth mode showed an N-shaped relationship, which indicated that before the first inflection point, urbanization development will aggravate marine pollution; between the first inflection point and the second inflection point, urbanization promotion will improve marine pollution; and after the second inflection point, urbanization development will increase marine pollution. Compared with the results of the spatial panel GS2SLS model, the relationship between urbanization depth model and marine pollution in the eastern marine economic circle conformed to the U-shape, but the urbanization breadth model did not. Therefore, consistent with the above research conclusions of national coastal cities, the impact of urbanization in the eastern marine economic circle on marine pollution is largely determined by its depth mode. One possible reason for this is that the eastern marine economic circle is the region with the fastest economic development and the highest urbanization level among the three marine economic circles, and the degree of agglomeration is also the largest. The resulting crowding effect aggravates marine pollution [66]. In terms of the inflection point, as for the urbanization depth mode, all cities in the eastern marine economic circle crossed inflection points at the end of the investigation period and entered the rising stage of the U-shaped curve. That is to say, marine pollution gradually increased with the development of urbanization depth. As for the urbanization breadth mode, all cities in the eastern marine economic circle crossed the first inflection point. However, two cities (Yancheng and Zhoushan) did not cross the second inflection point and are still in the middle stage of the N-shaped curve. The remaining cities crossed the second inflection point and entered into the rising phase of the N-shaped curve.

The above analysis of the national region and the eastern marine economic circle has drawn a grim reality. On the whole, the different urbanization patterns of China’s coastal cities and marine pollution are in a positive correlation stage. The coastal urbanization and marine pollution have not been “decoupled”, and coastal areas will face trade-offs between urbanization and marine pollution prevention. This result is similar to that of Chen et al. [35], who argued that marine economic growth and marine pollution in China have not been “decoupled” in recent years. Hence, we still have a long way to go before we can achieve the dual goals of urbanization development and marine environmental pollution improvement.

### 4.2. Mechanism Analysis Results and Discussion

The above analysis shows that urbanization of most coastal cities in China will aggravate marine environmental pollution. Therefore, for the green and sustainable development of coastal cities, we explored the mechanisms by which urbanization affects marine pollution. The regression results are shown in Table 8. Model (1), (2), and (3) represented the mechanism analyses of technological innovation, financial development, and human capital, respectively. Furthermore, the three models were estimated using 3SLS method.

As shown in Table 8, for the models (1), (2) and (3), the estimated coefficients of economic growth (lnpgdp) and urbanization (lnurban) were both significantly positive. However, the estimated coefficients of technological innovation (lnti), financial development level (lnfin), and human capital (lnhc) were significantly negative. Furthermore, the coefficient of the human capital effect was the largest, the coefficient of the financial development effect was second, and the coefficient of the technological innovation effect was the smallest. A possible reason is that China’s coastal cities have a strong attraction for high-level labor, but they are excessively dependent on technology introduction, and the financial development is not perfect [52]. Specifically, for model (1), continuous urbanization development can improve urban technological innovation level, so as to increase production efficiency [75] and promote cleaner production, ultimately improving marine environmental pollution in coastal cities. For model (2), urbanization boosts technological upgrading and promotes the upgrading of industrial structure by improving financial development level, thereby reducing the emission level of marine pollutants [53]. For model (3), with the promotion of urbanization, the improvement of human capital makes it not only easy to apply new technologies to boost natural resources utilization efficiency, but also can enhance the awareness of environmental protection [76,77], which promotes the decline of marine pollution. It can be concluded that urbanization can decrease marine pollution by improving technological innovation, financial development, and human capital. Thus, we should give full play to the functions of these three aspects so as to better reduce marine pollution in the process of urbanization.

## 5. Conclusions

Under the strategic background of building a “maritime power” and sustainable development, the increasingly serious marine pollution affects high-quality economic development and coastal residents’ health. Hence, it is of great significance to explore the relationship between urbanization and marine pollution for the sustainable development of coastal areas. Based on the concentration data of marine pollutants and NTL data of 46 coastal cities from 2006 to 2015, we used the GS2SLS method to discuss the impact of urbanization on marine pollution and the heterogeneity of regional and urbanization patterns under spatial effect and utilized the 3SLS method to analyze the mechanism between them. The main conclusions are as follows: (1) China’s marine pollution has a strong spatial spillover effect, and the relationship between coastal urbanization and marine pollution is U-shaped. At this stage, coastal urbanization has far more promotion effects on marine pollution than inhibitory effects. (2) The regional heterogeneity analysis showed that the relationship between urbanization and marine pollution in the eastern and southern marine economic circles is U-shaped, while the relationship in the northern marine economic circles is inverted U-shaped. The eastern and southern marine economic circles with serious marine pollution are in the phase of increasing marine pollution. (3) The heterogeneity of urbanization patterns indicates that for national coastal cities and the eastern maritime economic circle, the impact of urbanization on marine pollution is largely determined by the depth mode. The impact of the urbanization depth model and urbanization breadth model on marine pollution is in a positive correlation stage, and coastal urbanization development and marine pollution have not been “decoupled”. (4) Further mechanism tests showed that the technological innovation effect, financial development effect, and human capital effect can effectively reduce marine pollution in the process of urbanization. Moreover, in terms of the effect degree, the human capital effect is the largest, the financial development effect is the second, and the technological innovation effect is the smallest.

## 6. Policy Implications

Based on the above conclusions, this paper puts forward the following policy implications. Firstly, coastal cities need to further strengthen regional cooperation, break administrative boundaries, and realize joint prevention and governance. Moreover, the obligations and responsibilities in marine pollution control must be clarified, and policies and regulations on marine pollution control should be jointly formulated to regulate the worst-hit areas of marine pollution. Secondly, local government should efficiently use and plan land resources to improve the actual efficiency of urban space construction and reasonably project the urban layout. Furthermore, coastal cities should prevent and reduce the inefficient phenomenon of “environmental congestion” and give full play to the positive externality effect. Moreover, the marine pollution in the eastern and southern marine economic circles should mainly be curbed, and coastal areas should choose the appropriate urbanization development patterns according to their own actual situations. Thirdly, coastal cities should promote the transformation of economic and industrial structure and improve the efficiency of marine environmental governance. In addition, the environmental access threshold of FDI must be raised, and government intervention should be appropriately strengthened in controlling marine pollution. Finally, coastal cities should make comprehensive use of various ways to reduce marine pollution and realize the “decoupling” between economic development and marine environmental pollution. On the one hand, coastal cities should increase investment in technological innovation and accelerate the research and application of cleaner production technology and equipment. On the other hand, financial resources should be invested in green enterprises to develop green credit. At the same time, the talent strategy of “internal training and external introduction” should be implemented to form a human capital structure dominated by high-level human capital.

Although this study analyzes the impact and mechanism of urbanization on marine pollution, the following deserve further study in the future. Firstly, the asymmetric geography–economy weight matrix can be used to better describe the unequal spatial spillover effects between cities. Secondly, the spatial econometric analysis in this paper only attempts the GS2SLS model, so other models can be applied in the future, such as the spatial Durbin model. Finally, in the future, research on the transmission mechanisms of urbanization and marine pollution can be deepened, such as exploring the role of industrial structure in the impact of urbanization on marine pollution.

## Figures and Tables

**Table 1:** Descriptive statistics.

Variable Type	Variable Name	Symbol	Obs	Mean	S.D.	Min	Max
Explained variables	Marine pollution	lnmp	460	−1.910	0.660	−3.743	−0.017
Explanatory variables	Urbanization	lnurban	460	0.977	2.370	−6.048	7.117
	Quadratic term of urbanization	${\left(lnurban\right)}^{2}$	460	6.558	9.886	0.001	50.652
Control variables	Economic growth	lnpgdp	460	10.635	0.624	8.869	13.056
	Quadratic term of economic growth	${\left(lnpgdp\right)}^{2}$	460	113.482	13.276	78.656	170.451
	Population density	lnpop	460	6.298	0.546	4.890	7.882
	Energy efficiency	lnee	460	11.992	0.582	10.599	13.653
	Industrial structure	lnis	460	−0.720	0.210	−1.648	−0.215
	Degree of openness to the outside world	lnfdi	460	−3.843	0.989	−6.650	−2.028
	Government intervention	lngi	460	−2.418	0.371	−3.405	−1.494
	Marine economic development	lnpgop	460	8.746	0.721	6.457	10.410
Intermediate variables	Technological innovation	lnti	460	0.725	1.895	−5.088	6.362
	Financial development level	lnfin	460	0.296	0.365	−0.611	1.370
	Human capital	lnhc	460	0.116	1.122	−2.221	2.483

**Table 2:** Global Moran’s I of marine pollution from 2006 to 2015.

Year	2006	2007	2008	2009	2010
Moran’s I	0.365	0.457	0.184	0.405	0.439
*p*-value	0.008	0.001	0.134	0.003	0.001
**Year**	**2011**	**2012**	**2013**	**2014**	**2015**
Moran’s I	0.335	0.436	0.430	0.445	0.581
*p*-value	0.008	0.001	0.001	0.001	0.000

**Table 3:** Spatial GS2SLS model regression results.

Variables	(1)	(2)	(3)	(4)
	FE	RE	FE	RE
$W_{1}*lnmp$	0.085 ** (0.033)	0.056 ** (0.026)	0.102 *** (0.032)	0.077 *** (0.026)
lnurban	0.012 * (0.007)	0.009 * (0.005)	0.018 * (0.009)	0.016 ** (0.008)
${\left(lnurban\right)}^{2}$	0.011 *** (0.004)	0.012 *** (0.003)	0.009 ** (0.004)	0.011 *** (0.003)
lnpgdp	−0.738 *** (0.182)	−0.845 *** (0.163)	−0.833 *** (0.203)	−0.967 *** (0.179)
${\left(lnpgdp\right)}^{2}$	0.034 *** (0.010)	0.041 *** (0.009)	0.041 *** (0.011)	0.047 *** (0.010)
lnpop	0.154 (0.117)	0.250 ** (0.096)	0.116 (0.124)	0.203 ** (0.102)
lnee	0.099 * (0.058)	0.096 * (0.054)	0.093 * (0.053)	0.089 ** (0.041)
lnis	−0.061 (0.172)	0.067 (0.157)	−0.200 (0.194)	0.088 (0.178)
lnfdi			0.043 * (0.024)	0.039 * (0.023)
lngi			−0.188 * (0.109)	−0.203 ** (0.099)
lnpgop			0.052 (0.088)	0.056 (0.074)
Constant	−0.080 (0.092)	−0.092 (0.148)	−0.080 (0.102)	−0.082 (0.154)
N	460	460	460	460
Wald test (p)	667.999 (0.000)	624.583 (0.000)	680.351 (0.000)	602.647 (0.000)
Inflection point (urban)	0.580	0.687	0.368	0.483
Hausman test (p)	51.308 (0.000)		16.230 (0.131)	

Note: *, **, and *** indicate significance at the 10%, 5%, and 1% levels, respectively. Figures in () are standard error; FE and RE represent fixed effect and random effect, respectively.

**Table 4:** The results of robustness tests.

Variables	Replace Urbanization Index		Displace Space Matrix		Replace Instrumental Variables		Change Sample Size	
	(1)	(2)	(3)	(4)	(5)	(6)	(7)	(8)
	FE	RE	FE	RE	FE	RE	FE	RE
$W_{1}*lnmp$	0.102 *** (0.032)	0.075 *** (0.026)			0.097 *** (0.033)	0.072 *** (0.027)	0.103 *** (0.033)	0.093 *** (0.027)
$W_{2}*lnmp$			0.944 *** (0.213)	0.894 *** (0.210)				
lnurban	0.080 * (0.043)	0.082 ** (0.041)	0.071 * (0.041)	0.082 ** (0.039)	0.060 * (0.034)	0.061 * (0.032)	0.016 * (0.009)	0.013 * (0.007)
${\left(lnurban\right)}^{2}$	0.044 *** (0.015)	0.049 *** (0.014)	0.039 *** (0.014)	0.054 *** (0.013)	0.044 *** (0.015)	0.050 *** (0.014)	0.008 ** (0.004)	0.010 *** (0.004)
N	460	460	460	460	460	460	440	440
Control variables	Yes	Yes	Yes	Yes	Yes	Yes	Yes	Yes
Hausman test (p)	13.830 (0.243)		23.529 (0.015)		13.830 (0.243)		17.805 (0.058)	

Note: *, **, and *** indicate significance at the 10%, 5%, and 1% levels, respectively. Figures in () are standard error; FE and RE represent fixed effect and random effect, respectively.

**Table 5:** Regression results of regional heterogeneity analysis.

Variables	Northern Marine Economic Circle		Eastern Marine Economic Circle		Southern Marine Economic Circle	
	(1)	(2)	(3)	(4)	(5)	(6)
	FE	RE	FE	RE	FE	RE
$W_{1}*lnmp$	−0.110 *** (0.032)	−0.018 *** (0.030)	0.251 *** (0.047)	0.226 *** (0.074)	0.031 (0.041)	0.047 (0.053)
lnurban	0.044 * (0.024)	0.042 ** (0.021)	−0.113 *** (0.036)	−0.002 (0.034)	0.029 ** (0.012)	0.032 * (0.017)
${\left(lnurban\right)}^{2}$	−0.042 ** (0.018)	−0.035 ** (0.016)	0.020 ** (0.008)	0.009 (0.006)	0.009 ** (0.004)	0.005 (0.004)
Constant	−1.036 (0.368)	−2.198 (0.780)	1.084 (1.271)	0.397 (0.332)	0.396 (0.290)	0.139 (0.114)
N	160	160	90	90	210	210
Control variables	Yes	Yes	Yes	Yes	Yes	Yes
Hausman test (p)	7.039 (0.796)		−112.252 (0.000)		115.003 (0.000)	

Note: *, **, and *** indicate significance at the 10%, 5%, and 1% levels, respectively. Figures in () are standard error; FE and RE represent fixed effect and random effect, respectively.

**Table 6:** Regression results of different urbanization patterns in national coastal cities.

Variables	Urbanization Depth		Urbanization Breadth	
	(1)	(2)	(3)	(4)
	FE	RE	FE	RE
$W_{1}*lnmp$	0.099 *** (0.032)	0.073 *** (0.026)	0.100 *** (0.032)	0.076 *** (0.026)
lnurban1	0.121 ** (0.046)	0.127 *** (0.045)		
${\left(lnurban1\right)}^{2}$	0.050 *** (0.015)	0.055 *** (0.014)		
lnurban2			−0.015 * (0.008)	−0.029 * (0.015)
${\left(lnurban2\right)}^{2}$			0.026 ** (0.012)	0.034 *** (0.013)
Constant	−0.084 (0.102)	−0.085 (0.154)	−0.065 (0.101)	−0.067 (0.154)
N	460	460	460	460
Control variables	Yes	Yes	Yes	Yes
Inflection point (urban)	0.298	0.315	1.334	1.532
Hausman test (p)	9.990 (0.531)		19.674 (0.050)	

Note: *, **, and *** indicate significance at the 10%, 5%, and 1% levels, respectively. Figures in () are standard error; FE and RE represent fixed effect and random effect, respectively.

**Table 7:** Regression results of different urbanization patterns in eastern marine economic circle.

Variables	Urbanization Depth		Urbanization Breadth			
	(1)	(2)	(3)	(4)	(5)	(6)
	FE	RE	FE	RE	FE	RE
$W_{1}*lnmp$	0.212 *** (0.078)	0.269 *** (0.051)	0.215 *** (0.073)	0.212 *** (0.044)	0.270 *** (0.073)	0.246 *** (0.045)
lnurban1	0.001 (0.025)	−0.093 ** (0.043)				
${\left(lnurban1\right)}^{2}$	0.044 (0.027)	0.107 *** (0.038)				
lnurban2			−0.022 (0.062)	−0.211 *** (0.051)	−0.08 ** (0.039)	−0.269 *** (0.057)
${\left(lnurban2\right)}^{2}$			0.016 (0.015)	0.046 ** (0.021)	−0.005 (0.180)	0.014 (0.025)
${\left(lnurban2\right)}^{3}$					0.013 ** (0.006)	0.017 ** (0.007)
constant	0.320 (0.342)	0.617 (0.859)	0.339 (0.290)	1.185 (1.535)	0.388 (0.279)	1.053 (1.252)
N	90	90	90	90	90	90
Control variables	Yes	Yes	Yes	Yes	Yes	Yes
Inflection point (urban)	0.989	0.648	1.989	9.91	0.270/4.788	0.087/8.787
Hausman test (p)	−16.717 (0.117)		−184.966 (0.000)		−330.604 (0.000)	

Note: ** and *** indicate significance at the 5% and 1% levels, respectively. Figures in () are standard error; FE and RE represent fixed effect and random effect, respectively.

**Table 8:** The results of mechanism analysis.

Variables	(1)			(2)			(3)		
	lnmp	lnti	lnurban	lnmp	lnfin	lnurban	lnmp	lnhc	lnurban
lnti	−0.772 * (0.463)								
lnfin				−0.967 ** (0.391)					
lnhc							−1.353 * (0.805)		
lnurban		0.295 ** (0.125)			0.022 ** (0.011)			0.168 ** (0.079)	
lnpgdp			1.045 *** (0.243)			1.045 *** (0.243)			1.045 *** (0.243)
N	460	460	460	460	460	460	460	460	460
R^2^	0.702	0.970	0.933	0.855	0.970	0.933	0.707	0.965	0.933

Note: *, **, and *** indicate significance at the 10%, 5%, and 1% levels, respectively. Figures in () are standard error.

## Data Availability

The data shown in this research are available upon request.

## Appendix A

**Table A1:** Summary of main relevant literature based on the Chinese case.

Author(s)	Pollution Indicators	Methods	Data	Main Results
Liu, X. Sun, T. Feng, Q. [22]	Environmental pollution composite index	Dynamic spatial Durbin model	China; 2006–2017 Provincial-level statistics	There is no EKC between urbanization and environmental pollution in China, and urbanization will increase local environmental pollution.
Yu, B. [26]	Comprehensive pollution emission index	Dynamic spatial panel model	China; 2003–2017 Provincial-level statistics	China’s new-type of urbanization has not only effectively reduced pollution emissions and improved energy efficiency but has also been significant in terms of its ecological effects.
Liang, W. Yang, M. [32]	Regional wastewater discharge	Urbanization economic growth model and simultaneous equation model	China; 2006–2015 Provincial-level statistics	Environmental pollution has a significant inhibitory effect on urbanization; and there is an inverted U curve between urbanization and environmental pollution.
Shao, Q. Guo, J. Kang, P. [9]	Per capita volume of industrial wastewater discharged directly into the sea	Panel vector autoregressive model	China; 2000–2016 Provincial-level statistics	Marine economic and urbanization lead to marine pollution. Moreover, urban expansion aggravates marine environmental damage.
Chen, J. Wang, Y. Song, M. Zhao, R. [35]	Red-tide disaster areas by coastal region	Tapio elasticity coefficient method	China; 2002–2013 Provincial-level statistics	The research reaches an inverted N-shaped relationship between the marine economy and marine pollution in China.
Shao, Q. [8]	Industrial wastewater discharged directly into the sea	Panel threshold model	China; 2000–2016 Provincial-level statistics	The results reveal that the increase in per capita GOP strongly promotes marine pollution across the three phases of the panel threshold model, implying that China is still located in the first half of the EKC, before the peak.

Note: The order of literature in Table A1 is consistent with that in the paper.
